# Low Prevalence of Isolated Growth Hormone Deficiency in Patients After Brain Injury: Results From a Phase II Pilot Study

**DOI:** 10.3389/fendo.2018.00723

**Published:** 2018-12-17

**Authors:** Miriam Leonhardt, Anna Kopczak, Barbara Schäpers, Janina Limbrock, Philipp G. Sämann, Michael Czisch, Nicole von Steinbuechel, Martina Jordan, Harald J. Schneider, Manfred Schneider, Caroline Sievers, Günter K. Stalla

**Affiliations:** ^1^Schön Klinik Bad Aibling, Bad Aibling, Germany; ^2^Max Planck Institute of Psychiatry, Munich, Germany; ^3^Institute for Medical Psychology and Medical Sociology, University Medicine Göttingen, Göttingen, Germany; ^4^ClinSupport, Erlangen, Germany; ^5^Center for Endocrinology and Metabolism Nymphenburg, Munich, Germany; ^6^Institute of General Medicine, University Hospital of the Ludwig-Maximilians-University, Munich, Germany

**Keywords:** growth hormone, brain injury, neurorehabilitation, cognition, health-related quality of life, depression, visceral fat

## Abstract

Growth hormone deficiency (GHD) results in an impaired health-related quality of life (HrQoL) and cognitive impairment in the attention and memory domain. GHD is assumed to be a frequent finding after brain injury due to traumatic brain injury (TBI), aneurysmal subarachnoid hemorrhage (SAH) or ischemic stroke. Hence, we set out to investigate the effects of growth hormone (GH) replacement therapy in patients with isolated GHD after brain injury on HrQoL, cognition, and abdominal fat composition. In total, 1,408 patients with TBI, SAH or ischemic stroke were screened for inclusion. Of those, 54 patients (age 18–65 years) were eligible, and 51 could be tested for GHD with GHRH-*L*-arginine. In 6 patients (12%), GHD was detected. All patients with isolated GHD (*n* = 4 [8%], male, mean age ± SD: 49.0 ± 9.8 years) received GH replacement therapy for 6 months at a daily dose of 0.2–0.5 mg recombinant GH depending on age. Results were compared with an untreated control group of patients without hormonal insufficiencies after brain injury (*n* = 6, male, mean age ± SD: 49.5 ± 13.6 years). HrQoL as well as mood and sleep quality assessed by self-rating questionnaires (Beck Depression Index, Pittsburgh Sleep Quality Index) did not differ between baseline and 6 months within each group or between the two groups. Similarly, cognitive performance as assessed by standardized memory and attention tests did not show significant differences within or between groups. Body mass index was higher in the control vs. the GH replacement group at baseline (*p* = 0.038), yet not different at 6 months and within groups. Visceral-fat-by-total-fat-ratio measurements obtained from magnetic resonance imaging in 2 patients and 5 control subjects exhibited no consistent pattern. In conclusion, this single center study revealed a prevalence of GHD of about 12% (8% with isolated GHD) in brain injury patients which was lower compared with most of the previously reported cohorts. As a consequence, the sample size was insufficient to conclude on a benefit or no benefit of GH replacement in patients with isolated GHD after brain injury. A higher number of patients will be necessary to draw conclusions in future studies.

**Clinical Trial Registration**: www.ClinicalTrials.gov, identifier NCT01397500.

## Introduction

Pituitary insufficiency is assumed to be a frequent finding after traumatic brain injury (TBI) and subarachnoid hemorrhage (SAH) (1–4). In a systematic review of 19 studies that included 1,137 patients, the pooled prevalences of hypopituitarism in the chronic phase after TBI and SAH were 27.5 and 47%, respectively (2). Also after ischemic stroke, hypopituitarism is a prevalent condition. In one study pituitary dysfunction was detected in 37.5% of patients with ischemic stroke (1). Previously we have shown that neuroendocrine disturbances often persist over years after TBI or SAH (5) with the highest prevalence being observed 1–2 years post-injury. However, transient deficiency is also a known phenomenon, and patients may potentially recover from gonadotropic and somatotropic insufficiency (6).

Among the different disturbances, growth hormone deficiency (GHD) is the most frequent neuroendocrine abnormality after TBI and SAH (7). Clinical symptoms of GHD and signs often are unspecific and similar to the sequelae of brain damage itself and thus often remain undetected in brain injured patients (8).

In patients with pituitary adenomas, GHD has been shown to be associated not only with major changes in body composition such as diminished muscle strength and loss of bone mass, but also with impaired health-related quality of life (HrQoL) and cognitive deficits (9, 10). In addition, growth hormone (GH) has received attention as a possible general treatment directed toward repair of neural injuries, mainly due to its ability to promote neurogenesis in response to brain damage (11). Recombinant injected GH reaches the cerebrospinal fluid in GH deficient humans (12) from where it likely exerts its direct effects in the central nervous system. In animal models, treatment with GH *in vitro* (11) fosters the proliferation of hippocampal stem cells, and similarly, administration of IGF-1 *in vivo* has been reported to induce neurogenesis in the adult rat hippocampus (13). Furthermore, a neuroprotective role of GH, at least in part be mediated by IGF-1, has been suggested (14).

Preliminary evidence indicating that patients may benefit from GH replacement during neurorehabilitation in terms of HrQoL and cognition comes from small studies (15–20) and case reports (21–23). Table 1 summarizes these studies including the applied definition of GHD, GH dosage for substitution therapy, follow-up time, and key results. Two recent case reports suggest that improvements in memory and other cognitive domains as well as physical functions may even occur in the absence of pituitary dysfunction (22, 23). GH deficient patients in neurorehabilitation might therefore benefit from GH replacement or treatment. However, due to the overlap of symptoms of brain injury and hormone deficiency, it is not clear to what extent hormone deficiency actually contributes to the clinical picture and whether hormone replacement would be beneficial (24).

**Table 1:** Studies investigating GH replacement therapy in adult GH deficient TBI patients.

**References**	**Pts. (*N*)**	**Age of treated GHD patients (years)**	**GHD testing**	**Study design**	GH dose/ day^*^	**Follow-up**	**Parameters**	**Key results**
High et al. (16)	12 treated/11 controls	36.1 ± 10	Glucagon stimulation test	Open, prospective, randomized study	0.6 mg (or uptitrated)	12 months	Cognition: neuropsych. battery	Improvements in Dominant Hand Finger Tapping Test, WAIS III–PSI, CVLT II, WCST (executive functioning)
Maric et al. (19)	4 treated/2 controls (≥3 yrs after TBI)	39.3 ± 11	GHRH + GH releasing peptide-6	Open, prospective, study	0.3 mg (males), 0.4 mg (females)	6 months	Cognition: semi-structured interviews, SCL-90-R, ZDI, neuropsych. battery	Improvements in cognition, particularly of memory, and psychiatric functioning; worsening of memory and symptoms in 3 SCL dimensions after discontinuing GH therapy for 12 months (*n* = 3)
Reimunde et al. (17)	11/19 GHD, 8/19 controls w/o GHD	53.4 ± 17.4	GHRH-arginine	Open, prospective study, placebo-controlled study	1 mg	3 months	Cognition WAIS test	GHD: significant improvements in understanding, digits, numbers, incomplete figures, similarities, vocabulary, verbal and total IQ; GHD vs. controls: significant improvements in similarities, vocabulary, verbal and total IQ
Moreau et al. (15)	23 (27 controls)	37.9 ± 11.7	ITT or GHRH-arginine	Open, prospective, controlled study	0.2–0.6 mg	12 months	Cognition, HrQoL, ADL	Significant cognitive improvements, in particular for the ROCF, and in 2/6 QoL domains (personal, functional); greatest improvements if lower pre-treatment performance; modest correlation with ADL
Devesa et al. (20)	5/13 GHD (2.5 months−11 years after TBI)	24.6 ± 9.6	GHRH-arginine	Open, prospective study	0.8–1 mg/ 5 days/week, resting 15 days every 2 months	max. 8 months	Cognition, motor symptoms	Significant improvements in all patients; cognitive improvements appeared earlier and were more important than motor improvements; visual performance ameliorated in a patient with amaurosis
Gardner et al. (18)	161	42.6 [40.8; 44.5]	ITT or glucagon stimulation or GHRH-arginine or IGF-1 SDS	Retrospective database analysis	0.37 mg (95% CI, 0.35, 0.40)	8 years	HrQoL: QoL-AGHDA	Significant improvement of QoL-AGHDA score was sustained over 8 years; normalization of socialization after 1 year, self-confidence and tenseness after 6 years; no normalization of tiredness and memory

* *After titration period*. *ADL, Activities of Daily Living; AGHDA, Assessment of GH Deficiency in Adults; CI, confidence interval; CVLT, California Verbal Learning Test; GHD, growth hormone deficiency; GHRH, growth hormone releasing hormone; HrQoL, health-related quality of life; ITT, insulin-tolerance test; PSI, Processing Speed Index; ROCF, Rey-Osterrieth Complex Figure Test; SCL, Symptom-checklist; WAIS, Wechsler Adult Intelligence Scale; WCST, Wisconsin Card Sorting Test; ZDI, Zung Depression Inventory*.

The aim of the present clinical phase II study was to investigate whether stable patients with isolated GHD after brain injury do benefit from GH replacement therapy during neurorehabilitation. For this purpose, changes in HrQoL and cognition as well as of body fat composition following GH replacement therapy over 6 months in patients after brain injury due to TBI, SAH, or ischemic stroke were compared with brain injured patients without pituitary insufficiency.

## Materials and Methods

### Patients

All patients were attending rehabilitation therapy due to a recent event or repeated in-patient neurorehabilitation in the Schön Klinik Bad Aibling, Germany, which is a specialized clinical center for neurology and neurorehabilitation. Stable patients were screened from January 2012 to March 2015 in all phases of rehabilitation, i.e., the post-acute or chronic phase after TBI, aneurysmal SAH or ischemic stroke, excluding those on intensive care unit. Hormonal assessment was performed at least 1 month after the event or later. Main inclusion criteria were age 18–65 years, TBI of all grades, aneurysmal SAH of all grades or ischemic stroke. Before starting GH treatment, other hormonal axes had to be sufficient or treated sufficiently with a stable substitution therapy.

Exclusion criteria were pregnancy and lactation period, woman of childbearing potential not using an adequate method of birth control, men who are not willing to use an adequate method of birth control, previous or concomitant medication with GH, suspected or known hypersensitivity to GH treatment, substance abuse, any condition which in the opinion of the investigator makes the patient unsuitable for inclusion, participation in another clinical trial with an investigational new drug, planned treatment or changes in established treatment with any other drug which might significantly influence the GH axis or cognitive function (e.g., treatment with antidepressants), non-ability to perform testing, presence of any other condition listed in the contraindications or warnings in the local Summary of Product Characteristics of GH, and onset of GHD before brain injury.

Assumed α = 0.05 and β = 0.2, the necessary sample size calculated for this pilot study was 8 patients to detect HrQoL scores before and after GH replacement that are at least 1.3 fold the pooled standard deviation. We aimed to include at least 9 patients and 9 controls without pituitary insufficiency considering a drop-out rate of 10%.

All patients enrolled granted their written informed consent to participate in this pilot study in accordance with the Declaration of Helsinki. The study was approved by the ethics committee of the Bavarian Chamber of Physicians.

### Study Design and Treatments

This was an open phase II pilot study to investigate the influence of GH replacement therapy over 6 months on quality of life, cognition, body mass index (BMI), and abdominal fat distribution of GH deficient patients in stable, chronic phase after brain injury (EudraCT No: 2010-020679-21) in comparison with patients without pituitary insufficiency and no GH replacement treatment. Patients with isolated GHD received open treatment with recombinant human GH SC (Genotropin®, Pfizer Pharma GmbH). Since GH requirements decrease with age and are higher in women than in men (25), the following daily doses were applied: men <45 years; 0.4 mg; ≥45 years: 0.2 mg; women <45 years 0.5 mg, ≥45 years: 0.3 mg. All GH deficient patients started with the half dose for the first 4 weeks. A follow-up visit was scheduled at 8 weeks after starting GH replacement therapy. A third study group not included in this report included male patients with hypogonadism.

The primary endpoint of the study was to evaluate changes in quality of life in GH deficient patients before and 6 months after GH replacement treatment and in comparison to control patients. Secondary endpoints included changes in cognition scores, BMI, and abdominal fat distribution within and between the study groups.

### Assessments

Basic medical history included causes and grading of TBI according to the Glasgow Coma Scale (26) as well as localization of SAH and its evaluation according to Hunt & Hess (27), the World Federation of Neurosurgical Societies (27) and Fisher scales (28).

According to the study protocol, both the insulin tolerance test (ITT) and the GH releasing hormone (GHRH)-*L*-arginine test were allowed to assess for GHD. The primary aim was to use the ITT as gold standard to stimulate the hypothalamic-pituitary GH axis. However, due to patient′s preferences and frequent contraindications such as seizures, diabetes or heart pain in the medical history of our patient population, all patients were not tested for GHD with the ITT but with the GHRH-*L*-arginine test. The GHRH-*L*-arginine test was performed as described before (29). In short, a GHRH bolus (1 μg/kg) was injected IV, followed by an infusion of arginine hydrochloride (0.5 g/kg body weight) over 30 min. Blood was drawn 30, 45, 60, 90, and 120 min after injection and the GH peak was determined. GHD was diagnosed dependent on BMI values (BMI < 25 kg/m^2^: cut-off ≤ 11.5 ng/mL; BMI ≥25 to < 30 kg/m^2^: cut-off ≤ 8 ng/mL; BMI ≥30 kg/m^2^: cut-off ≤ 4.2 ng/mL) (30).

Basal hormone levels such as free thyroxine (fT_4_) for the thyroid axis (normal range 0.93–1.7 ng/dL [11.97–21.88 pmol/L]), fasting cortisol (normal range 5–25 μg/dL [0.14–0.69 μmol/L]), and testosterone in men (normal: ≥3.5 ng/mL [≥12.1 nmol/L]) were measured in serum for screening. IGF-1 was measured 8 weeks after start of the therapy to adjust GH dosage as it is usually performed in the department of endocrinology at the Max-Planck-Institute of Psychiatry, Munich. All endocrine tests and blood drawings were performed between 7:45 and 8:15 a.m. after an overnight fast of at least 12 h. Quality of life and cognition were evaluated by using standardized instruments described in Table 2. Changes in weight were evaluated by BMI, and changes in abdominal fat distribution by MRI based volumetry. In addition, patients were evaluated for tolerability of GH replacement therapy by assessing adverse events at every visit.

**Table 2:** Standardized assessment of quality of life and cognition.

**Evaluation of HrQoL, Sleep, and Depression**	
12-Item Short Form Health Survey (SF-12) (31)	12-item self-reported health status
EuroQoL (EQ-5D) (32)	Self-completion questionnaire on health outcome
Quality of Life after Brain Injury (QoLIBRI) (33, 34)	Assessment of health related QoL and disturbing, negative aspects specifically in brain-injured patients
Beck Depression Inventory (BDI) (35)	Common questionnaire to quantify signs of depression
Pittsburgh Sleep Quality Index (PSQI) (36)	Assessment of self-reported sleep quality
**Evaluation of cognition**	
Verbal Learning and Memory Test (VLMT) (37)	Test for serial learning and learning of listings tested twice with an interval of 30 min
Tests from the psychological TAP 2.3 test battery of attention (38)	• Alertness Test for reaction time • Test of Vigilance for concentration • Go/NoGo test for the patients' reaction within a period of 2 min, which is the representative time for decision making

### MRI Based Abdominal Fat Measurements

Abdominal MRI was acquired at baseline and month 6 in all 10 participants using a breath holding T1-sequence (2D fast spoiled gradient echo, in-plane resolution 512 × 512 points, field-of-view 44 × 44 cm^2^, breath hold acquisition with two imaging slabs of 20 s each, 2 × 25 slices, slice thickness 8 mm, slice gap 1 mm) on a 1.5 Tesla clinical MR system (General Electrics, Signa Xcite, Milwaukee, U.S.A.). Anonymized images were transferred to the ROI tool of BRUKER Paravision 6.0.1. Here, the border between the visceral and subcutaneous compartment was defined manually by a spline curve for each slice, for all slices from the lower boundary of the liver defined as the most superior slice. To determine the image intensity of the fat compartment, an intensity histogram of the slice with the highest average intensity value (which is an indication for containing fat) underwent histogram analysis that typically shows a bimodal distribution, with a lower peak round the intensity of inner organs and skeletal muscles, and a higher intensity representing the fat compartment. The cut-off intensity for automated segmentation was then set at 2/3 of the position of the second peak. Per slice, the total volume of fatty tissue was determined (TF; number of voxels >cut-off intensity), as well as the volume of fatty tissue inside the visceral ROI (VF) and the VF-by-total-fat ratio (VTFR). The latter VTF ratio was averaged between slice 7 and the last slice on which VF was reliably detected by the rater (between slice 38 and 42); the resulting VTFR_global_ was forwarded to quantitative analysis.

### Statistical Analysis

All statistical analyses were performed using SPSS statistics (Version 20.0, Chicago, USA, SPSS Inc.). To prove for normal distribution the Shapiro-Wilk test was applied, which is appropriate for small sample sizes. Due to non-normal distributed data the non-parametric Mann-Whitney-U test was used to analyse the differences between the treatment and control group. For comparison of the pre and post values within each group the Wilcoxon signed-rank test was used. In all tests, an alpha level of 0.05 was considered statistically significant.

## Results

### Patient Disposition and Characteristics

**Figure 1:**
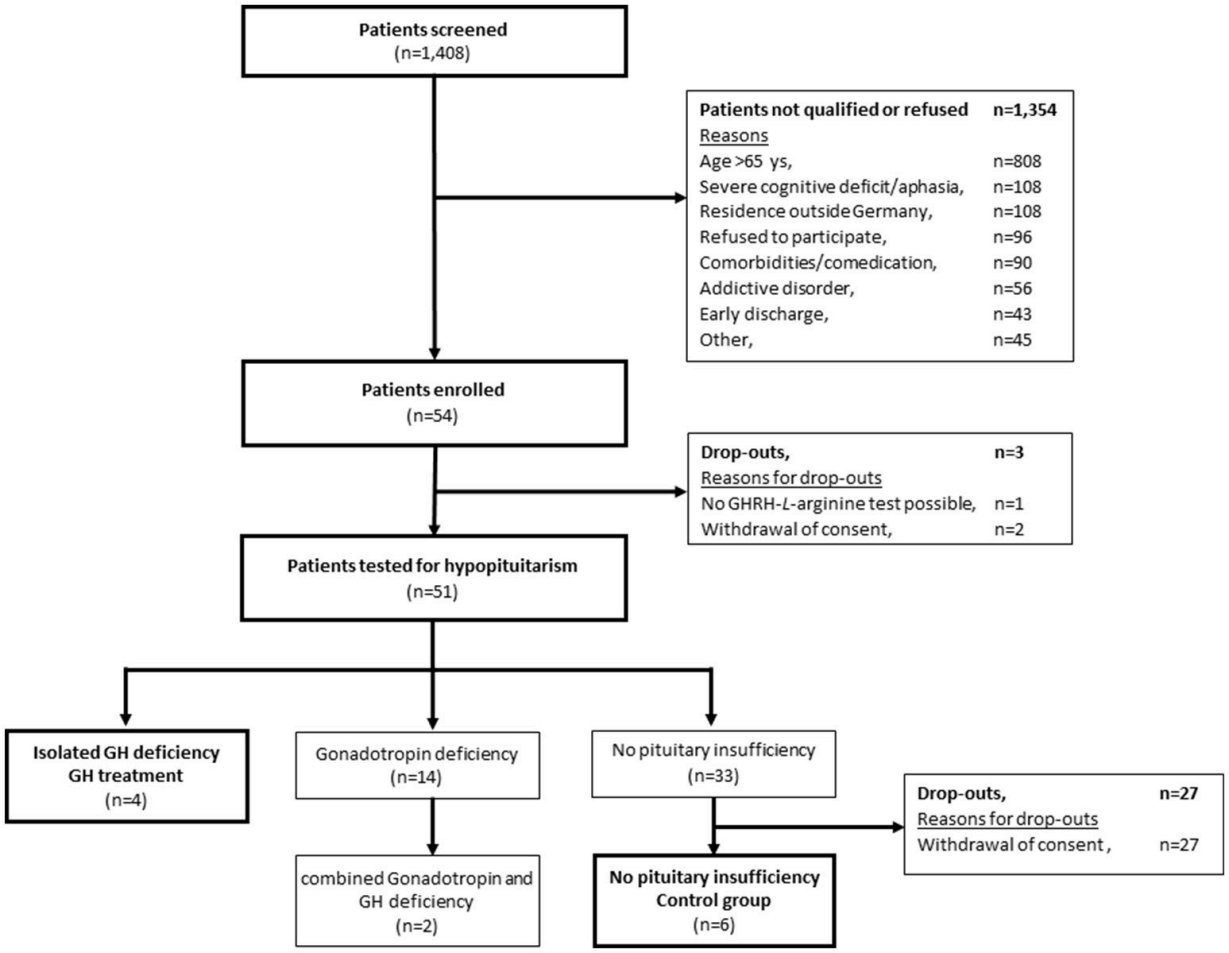
Patient disposition.

In total, 1,408 patients were screened. Of those, 54 patients met the inclusion criteria. 60% of the screened patients (*n* = 808) were older than 65 years old and thus could not participate in the study. 8% (*n* = 108) were not able to perform the neuropsychological test battery because of severe neurological and cognitive deficits according to the clinical impression. Eventually, 54 patients were enrolled in the study the first step of which was to test for GHD. Of the 54 patients included, hormonal assessment could not performed in 3 patients due to withdrawal of consent in 2 patients and technical failure in 1 other patient (Figure 1).

Thus, 51 of 54 patients underwent the GHRH*-L*-arginine test for GHD. In 6 of 51 successfully tested patients (12%) GHD was detected. In 2 of these patients, additional testosterone deficiency was detected which disqualified them for participation in this study. 33 of the 51 successfully tested patients (88%) did not present with any pituitary insufficiency.

All 4 patients with isolated GHD were included into the study and underwent GH replacement. Their mean age ± SD was 49.0 ± 9.8 years. One of these patients had TBI, 2 patients had aneurysmal SAH, and 1 patient had ischaemic stroke (Table 3).The control group consisted of 4 patients with ischemic stroke, 1 SAH patient, and 1 TBI patient without proven pituitary insufficiency. Mean age ± SD of the control group was 49.5 ± 13.6 years. All patients included were male.

**Table 3:** Baseline characteristics of individual patients.

**Lesion**	**Sex**	**Age (years)**	**Time to injury (days)**	**Severity**	**BMI (kg/m^**2**^)**
**GHD GROUP**, ***n*** **=** **4**					
Ischemic infarction	Male	51	25	NA	24.5
TBI	Male	40	78	Grade III	23.3
SAH	Male	62	94	Fischer grade II, WFNS grade I	21.9
SAH	Male	43	1,024	Hunt & Hess grade IV; Fischer grade III	25.6
**CONTROL GROUP**, ***n*** **=** **6**					
SAH	Male	56	63	Hunt & Hess grade III; Fischer grade III	27.2
Ischemic infarction	Male	52	53	NA	29.6
Ischemic infarction	Male	54	41	NA	26.1
Ischemic infarction	Male	56	66	NA	23.4
Ischemic infarction	Male	57	2,566	NA	26.7
TBI	Male	22	132	Grade III	26.4

*GH, growth hormone; NA, not applicable; SAH, subarachnoid hemorrhage; TBI, traumatic brain injury; WFNS, World Federation of Neurosurgical Societies*.

### Changes in Patient-Reported Outcomes (PROS) and Performance-Based Outcomes (PERBOS): Health-Related Quality of Life and Cognition

In the group of GH deficient patients no significant changes in any of the HrQoL scores assessed were observed after 6 months of GH replacement therapy with respect to baseline scores (Table 4). The same was true for the group of control patients without showing significant changes of quality of life scores, with the only exception of an improved QoLIBRI score (*p* = 0.042). In addition, there were no significant differences in any test when comparing quality of life scores between the intervention and control groups at either baseline or 6 months (Table 4).

**Table 4:** Changes of quality of life scores in patients with GH replacement therapy and controls.

**Test**	**GH replacement**		**Control group**		***P*-values**
	Baseline	6 months	Baseline	6 months	Group comparisons: baseline vs. 6 mo (GH/control) Time comparisons: GH vs. control (baseline/6 mo)
**QoL**					
**SF-12**					
Physical	37.8 ± 11.4 (*n* = 4)	55.9 ± 1.3 (*n* = 2)	48.4 ±6.4 (*n* = 6)	46.3 ± 11.8 (*n* = 6)	NA/0.144 0.200/NA
Psychological	46.0 ± 9.9 (*n* = 4)	47.1 ± 15.3 (*n* = 2)	50.8 ± 8.8 (*n* = 6)	56.3 ± 8.2 (*n* = 6)	NA/0.465 0.686/NA
EQ-5D (VAS)	63.8 ± 26.9 (*n* = 4)	67.0 ± 30.7 (*n* = 4)	51.3 ± 8.9 (*n* = 6)	60.7 ± 7.7 (*n* = 6)	0.273/0.157 0.730/0.556
QoLIBRI	59.8 ± 18.1 (*n* = 4)	69.3 ± 21.5 (*n* = 4)	77.9 ± 19.2 (*n* = 5)	82.6 ± 17.5 (*n* = 5)	0.068/0.042 0.286/0.413
BDI	13.3 ± 5.0 (*n* = 4)	11.5 ± 7.0 (*n* = 4)	9.2 ± 6.8 (*n* = 5)	5.2 ± 5.0 (*n* = 5)	0.285/0.109 0.556/0.111
PSQ1	6.5 ± 3.3 (*n* = 4)	5.0 ± 3.7 (*n* = 4)	4.0 ± 1.7 (*n* = 5)	4.8 ± 4.1 (*n* = 5)	0.357/0.496 0.413/1.000

*GH, growth hormone; SF-12, Short Form 12; EQ-5D, EuroQoL-5D; QoLIBRI, Quality of life after brain injury; BDI, Beck Depression Inventory; PSQI, Pittsburgh Sleep Quality Index*.

Concerning the secondary endpoints of changes in the TAP subtests on Alertness, Go/NoGo and Vigilance as well the VLMT the results did not differ significantly either with time or between the two groups at the beginning or after the intervention (data not shown). Some of the patients reported on a subjective well-being after initiating a GH replacement therapy and asked for continuing the therapy at the end of the study.

### IGF-1 and BMI

In the GH replacement group IGF-1 values increased (mean ± SD) from 128.8 ± 17.3 ng/mL to 214 ± 39.9 ng/mL proving the effectiveness of replacement therapy. IGF-1 concentration in the control group at baseline were 216.7 ± 51.6 ng/mL.

The BMI did not change significantly in either study group. In the GH replacement group BMI was 23.8 ± 1.6 kg/m^2^ at baseline and 24.7 ± 1.6 kg/m^2^ at 6 months (*p* = 0.273). In the control group it was 26.6 ± 2.0 kg/m^2^ at baseline and 26.5 ± 3.0 kg/m^2^ at 6 months (*p* = 0.753). Baseline BMI was significantly higher in the control group compared to the GH replacement group (*p* = 0.038) but there was no significant difference in BMI between the 2 study groups at 6 months (0.352).

### Visceral-to-Total Abdominal Fat Ratio Analysis

**Figure 2:**
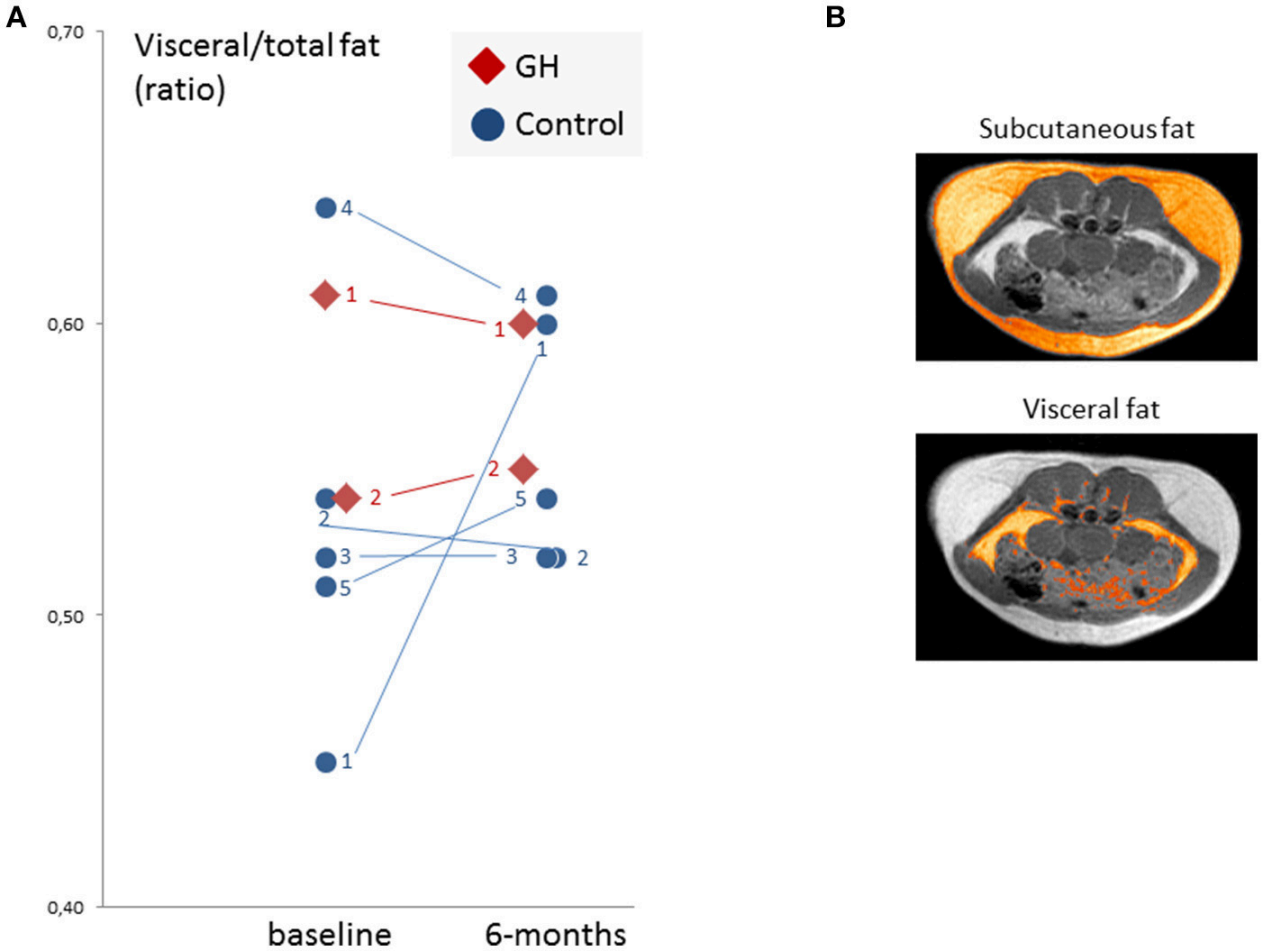
Abdominal fat measurement results. **(A)** Individual visceral/total fat ratio values of 2 GH and 5 controls at baseline and follow-up. Individual subject numbers are given. Eventually, no consistent or generalizable pattern emerged. **(B)** Exemplary axial slice (one of about 35 slices) with highlighted subcutaneous (upper) and visceral fat (lower) ROIs. Calculation of the fat compartment volumes was based on a semiautomated method requiring the manual definition of the inner border of the subcutaneous compartment and an absolute segmentation threshold gained from the typical bimodal intensity histogram of the slice with the highest total amount of fat (see section Materials and Methods for details).

Baseline and follow-up visceral-fat-by-total-fat-ratio (VTFR_global_) values were available for 2 GH subjects and 5 control subjects (see Figure 2), with no consistent pattern emerging. The control group was indifferent between both time points (*p* > 0.5), and the 2 GH patients also showed no deviating pattern (no statistics performed).

### Adverse Events

Adverse events were observed in all patients who were on GH replacement therapy consisting of diarrhea, itching, indurated thyroid, elevated IGF-1 level, confusion (all five adverse events in 1 patient), reddened throat (in 2 patients), as well as increased appetite and atopic eczema of the lower leg (each in 1 patient). Except for 1 patient who suffered from diarrhea due to infection with Clostridium difficile adverse events were not serious. All adverse events resolved and no serious adverse event was considered related to GH substitution. There was no death in either study group.

## Discussion

Our study confirms previous evidence that patients with brain injury due to TBI, SAH or ischemic stroke are at risk of developing GHD. However, in our study cohort, the prevalence of GHD was 12% and thus at the lower end of the range of prevalence when considering previous meta-analyses and large studies (1, 2, 39, 40). The prevalence of isolated GHD was even lower with only 8% of our screened patients. In contrast to others, we could not demonstrate significant improvements in HrQoL, cognition, and BMI following GH replacement therapy in GH deficient patients with brain injury.

In the past, posttraumatic hypopituitarism was considered to be a frequent finding. In a meta-analysis by Schneider et al. the pooled prevalence of GHD ranged between 7.9 and 36.7% in adult patients with aneurysmal SAH or TBI (2). Even higher prevalence rates were reported from the German Database on Hypopituitarism (*n* = 1,242). In this structured assessment, the prevalence of hypopituitarism in the chronic phase of patients with TBI and SAH by laboratory values, physician diagnoses, and stimulation tests was 35, 36, and 70%, respectively (5).

In the present, these high prevalence rates are raised to question. A recent review article by Klose et al. summarized prevalence of pituitary dysfunction from negligible to up to 70% in adults (41). In our patients using the GHRH-*L*-arginine test and BMI-dependent IGF-1 cut-offs the prevalence of isolated GHD amounted to 12%. This is in accordance with results such those of Klose et al. who found GHD in 12% of patients with moderate and severe TBI in the Danish National Study on Posttraumatic Hypopituitarism (*n* = 439 patients), also when testing with GHRH-*L*-arginine (42).

However, some endocrinologists require the proof of GHD using two different GH stimulation tests. Thus, GHD would only be considered when both tests reveal lowered stimulated GH values. This has also been investigated in the Danish cohort. GHD was only confirmed in 1% (and not in 12%) of patients when 2 tests identifying GHD were performed.

Applying both the ITT and the GHRH-*L*-arginine test in one patient may dramatically reduce the prevalence of GHD. In our study, both the ITT and the GHRH-*L*-arginine test were generally allowed, but due to the specific clinical profile of our patient cohort, only the GHRH-*L*-arginine test was applied; we did not confirm the presence of isolated GHD with a second stimulation test. Generally, the mentioned diversity in the prevalence rates due to different endocrine test assays including reproducibility, cut-off selection, assay heterogeneity, pre-test probability of hypopituitarism, and inappropriate reference population (40), may lead to two problems:

First, an over-conservative definition of the presence of a GHD after brain injury may falsely preclude patients from being enrolled in a GH study, despite a potential clinical benefit, which generally hampers the progress in answering the efficacy question. Beyond this, also the pathophysiological processes during recovery from brain injury with a potentially higher need of GH put into question, if standard cut-offs to diagnose isolated GHD are ideal to define the indication for a replacement treatment in brain injury patients. Possibly, also patients with the GH system working “at the normal limit” could benefit from GH replacement to foster neuronal recovery (43) and thus may be included as separate patient subgroup.

Second, due to the masked clinical presentation of GHD in brain injury patients, the indication for endocrine tests may be recognized too late in clinical routine, making prevalence rates unreliable until tested in large and heterogeneous brain injury populations with different endocrine tests to backtrack the effect of specific injury-related risk factors (such as brain injury location or type) and the endocrine cut-off criteria. Such refined epidemiological studies including elderly patients are essential to define the ideal patient population for GH replacement studies. In regard to the aging population the inclusion of elderly patients is necessary to reflect clinical settings. First insights suggest that elderly patients with GHD benefit from GH replacement showing improved cognitive function (44). Hence, elderly patients with GHD after brain damage should not be excluded from clinical studies investigating the effect of GH replacement on cognition. It should be taken into account that lower GH dosages are needed in elderly patients (25).

The best time window for GH treatment after brain injury is not known. Several studies included patients many years after brain injury (see Table 1). Devesa et al. reported about patients even 11 years after TBI. This may be due to the delay in diagnosis and the confounding symptoms that may occur as sequelae of brain injury as well as a symptom of GHD. Since GH replacement is an effective therapy reducing morbidity and mortality in GHD patients (45, 46), we did not want to exclude patients from this potentially useful therapy. Therefore, there was no upper limit for the time between brain injury and GH replacement. Hence, one of our patients suffered from brain injury even 2.8 years ago. But it can be discussed whether there is an optimal time window for replacement therapy, especially in regard to influence neuroplasticity after brain injury. GH is known to cross the blood-brain barrier (47); and its positive effects on neuroregeneration have been previously well-described (48). So it can be assumed that patients after brain injury may benefit from increased GH levels in the brain (43). In our study, all participants screened were in a stable phase after brain injury to avoid that (i) changes in hormonal levels are only due to acute adjustment effects and (ii) brain edema hampers GH to permeate through the blood-brain-barrier. There is no information available on GH levels in cerebrospinal fluid of our patients. To measure this, a lumbar puncture would have been necessary which would have increased the burden for participation in our study.

Moreau et al. (15) reported that patients after TBI showed improvement in cognition and quality of life after replacement therapy. The authors emphasize that this may be especially true for patients with severe disabilities. However, such a subgroup analysis was not possible due to the low sample size in our study cohort. Since brain pathology is not always related to the extent of cognitive deficits, we performed MRI scans and assessed cognitive functioning with neuropsychological tests.

Some limitations in our study deserve consideration. Although we screened a very large number of patients, only as few as 54 (3.8%) patients matched our study protocol and were willing to undergo GH-specific testing. Currently, GHD is not screened for in clinical routine; instead, the test was part of the study protocol and needed the patient‘s explicit consent which naturally reduces inclusion rates. Additional screening drop-outs were mainly due to age, but also due to severe cognitive deficits including aphasia, co-medication, co-morbidities, and refusal to participate.

For assessing HrQoL, time after brain injury can be crucial and differed between patients. GH und control group were rather heterogeneous in regard to diagnosis, age, and time since injury. However, one cannot be sure that small differences between groups can be ascribed to GHD.

Our study was designed to detect differences in cognition in patients after brain injury with GHD. Most of the applied neuropsychological tests are only validated up to the age of 65. Therefore, the age of our study participants was restricted to an upper limit of 65 years. Applying this criterion, as much as 57% of all screened patients were excluded from our study. In addition, patients had to be able to participate in a detailed neuropsychological assessment which not possible for 8% of the patients aged 18–65 years in neurorehabilitation. Less strict inclusion criteria would have probably allowed including more patients in our study. This implicates that patient selection directly affects the number of those who can potentially benefit from GH replacement. Moreover, the follow-up period of 6 months may have been too short to detect possible long-term effects in our cohort.

In contrast to other studies (15–19, 21–23) (Table 1), we could not show improvements in neither generic HrQoL, cognition nor body composition after GH replacement therapy in the expected direction. Ameliorated TBI-specific HrQoL was observed in the control group without pituitary insufficiency 6 months after baseline assessment. All mentioned results however have to be seen under the premise of the very small sample sizes and therefore reduced statistical power to detect significant and valid changes.

With regard to tolerability, our results may confirm that administration of GH is safe in brain injured patients (15, 18, 19). However, these results do also not allow for a definitive conclusion on the safety of GH replacement therapy in GH deficient patients after brain injury since all studies were small and rather heterogeneous in nature.

In conclusion, we have screened a very large number of patients after brain injury due to TBI, aneurysmal SAH, and ischemic stroke in real-world clinical practice. GHD was detected in 12% of all patients assessed with a stimulation test. In 8% of all tested patients, isolated GHD was diagnosed and study treatment with GH was initiated. At the end, we cannot confirm nor disprove a benefit of GH replacement in patients with isolated GHD after brain injury. However, this study is limited by the very small number of GH deficient patients that could be subjected to replacement therapy. *Summarized, due to only limited supporting evidence, patients with GHD after brain damage should–if at all–only receive GH replacement therapy upon an individual decision*. Larger, well-controlled studies are mandatory, that may also include placebo treatment, elderly patients, and probably also patients without measurable GHD.

## Author Contributions

ML was the study physician in Bad Aibling. She performed the stimulation tests and was the contact person for the study participants. AK was involved in the evaluation of the study design. At the beginning of the study, she was study physician in Bad Aibling. In the meantime she changed to the Max-Planck-Institute of Psychiatry where she was the contact person and study physician for healthy controls living in Munich. She prepared regulatory documents and wrote the manuscript. BS was the study nurse in Bad Aibling. She performed the screening of patients, prepared the stimulation tests and was a further contact person for the study participants. JL performed statistical analysis for this study. PS and MC performed and analyzed the MRI scans for visceral fat measurements and carefully revised the manuscript. NvS contributed with the Qolibri and gave advice on quality of life measurements including revision of the manuscript. MJ prepared all regulatory documents for this study. HS and MS were involved in the evaluation of the study design. CS was principal investigator and prepared regulatory documents. GS was responsible as sponsor of this study.

### Conflict of Interest Statement

This study was funded by an unrestricted grant from Pfizer Pharma GmbH unter grant agreement number WS478950. The study funders had no role in the study design, data collection and analysis, decision to publish, or preparation of the manuscript. MJ was employed by company ClinSupport. The sponsor GKS commissioned ClinSupport as a clinical research organisation to support the study team. CS received congress and lectures fees from Pfizer.
